# A Case of Visceral Disease, with an Enormous Hydatid, &c.

**Published:** 1823-10

**Authors:** W. Pretty

**Affiliations:** Member of the Royal College of Surgeons, &c.


THE LONDON

### Medical and Physical Journal.

4 OF VOL. L.] OCTOBER, 1823. [No. 296.

For many fortunate discoveries in medicine, and for the detection of numerous errors, the world is indebted to the rapid circulation of Monthly Journals; and there never existed any work, to which the Faculty, in Europe and America, were under deeper obligations, than to the Medical and Physical Journal of London, now forming a long, but an invaluable, series.—RUSH.

### ORIGINAL COMMUNICATIONS,

### SELECT OBSERVATIONS, &c.

ART. I.—*A Case of Visceral Disease, with an enormous Hydatid, &c.* By W. PRETTY, Esq. Member of the Royal College of Surgeons, &c.

THE following rather remarkable case occurred in the practice of my partner, Mr. Bagster, from whom, and the friends of the late patient, I have obtained the brief particulars of the symptoms during her severe illness. Dr. J. Johnson saw her several times while she was living; he was present at the examination of the body after death, with myself and our apprentice: I also assisted at the first paracentesis.

Mrs. N., thirty-two years of age, having been in very considerable practice as a midwife to a very extensive charity, was consequently subject to great fatigue of body and anxiety of mind. She was married at twenty, and had borne five children, two of which are now living, one eleven and the other five years old; one child died fourteen months old, and the other two were born dead. Four years ago her illness commenced with most agonizing pains in the head, and which state of suffering confined her nineteen weeks to her room, before she could obtain much relief. The pains were always more or less present to the hour of her death, but latterly a good deal eclipsed by sufferings produced from abdominal disease. During one portion of her illness, she was under a very respectable practitioner, who, either accidentally or designedly, threw her into a state of mercurial salivation, which was not followed by any mitigation of her pains. Mrs. N. was now so very much distressed by suffering, and very much harassed by her midwifery duties, which she would perform so long as she was in any way able to leave her house, that she determined to obtain admission into Guy's Hospital; where she was nine weeks confined with almost insupportable pains in the head, for which she was bled, blistered, and cupped repeatedly, with very little advantage over her disease: it was with great difficulty that she so far recovered as to return home.

It was a few weeks after this period that Mr. Bagster's attendance commenced, and it was continued through the last six months of her life. Mrs. N. had unfortunately indulged in a strong inclination for drinking ever since the birth of her last child; and, when Mr. B. first saw her, he found her with an enlarged liver, with ascites (as he then supposed), with anasarca of the lower extremities, and with pulmonary irritation attended by great difficulty of breathing, cough, and mucous expectoration; the pains in the head were scarcely spoken of. The usual remedies for dropsical affections were tried, and the lungs were in some degree relieved; but the swelling of the abdomen increasing, from the further accumulation of water, Mr. B. recommended her to be tapped. I was present at this operation, and felt the fluctuation of fluid very distinctly before the trocar was introduced; the abdomen also presented the usual even distention of ascites. The opening was effected in the linea alba, and half a pailful (about six quarts) of a greenish coloured fluid, was quickly discharged. The flow having ceased, we found, after withdrawing the canula, that the abdomen had not lost its size to the extent usual after tapping for that disease; yet no distinct tumor was to be felt, except the enlarged liver. Mrs. N. soon filled again, and nearly to the same size; and a second evacuation of the fluid by the use of the trocar was attempted by Mr. Bagster, about one month after the first operation. At this time only two pints of a greenish-coloured, ropy fluid were drawn off. A probe was introduced through the canula, to remove any obstructing body, but no further flow could be effected; consequently, her size suffered but little diminution. The fluid first evacuated by paracentesis acquired so much thickness by cooling, that it would scarcely pass through the drain-holes of a common sink; and that which was drawn off at the second operation became so very thick, by the same means, as to give a strong resemblance to calf's-foot jelly, with the exception of colour. A very severe purging of watery stools, with frequent vomiting, soon succeeded the last operation, and continued, in defiance of all remedies, for nearly a fortnight, when she at last sunk under her load of disease, early in July 1823.

*Post-mortem examination.*—The corpse was very much extenuated in the face and extremities, with the abdomen considerably enlarged, which, upon being struck, gave evidence of the fluctuation of a fluid; and the liver could be very distinctly traced as low down as the umbilicus. An incision from the upper part of the sternum to nearly the os pubis, with a cross incision towards the ilia, exposed the liver, and a firm white smooth substance, which concealed the whole of the intestines, and which much perplexed us at first as to its nature and formation: it was cut through longitudinally, and immediately a considerable quantity of a greenish-coloured fluid escaped, and large portions of a jelly-like substance were easily taken out of its cavity; and this substance was soft, and of somewhat cellular-like structure, for pressure in the hand caused it to yield very readily, and in abundance, the above-mentioned fluid. The fluid and the substance, when removed, nearly filled a common-sized wash-hand bason; consequently, a very considerable cavity was exposed; but, the sides of the cyst falling inwards, it was never seen in a distended state after the escape of fluid. The edges of the cyst were an eighth of an inch in thickness; and the cyst being very difficult of removal, from its very firm adhesions, was torn into several portions in effecting it. Upon closer examination, it proved an enormous hydatid, which many of my medical friends had an opportunity of seeing: it extended from the anterior inferior margin of the liver, to which it adhered nearly through its whole extent, and so firmly at one part as to require several strokes of the scalpel for its separation; over the surface of the other abdominal viscera, to the superior aperture of the pelvis, forming attachments to the peritoneum lining its sides, and also to that lining the abdominal muscles, yet leaving a surface anteriorly, four inches long and three wide, not attached: it required strong pressure of the fingers to separate these attachments; but from the surface of the intestines it was very easily peeled off, and most certainly it had no connexion with either the ovaria or the uterus. The shape of the hydatid may be likened to that of a distended urinary bladder, but with a finger-like process on the right side, and also a smaller one, which at first sight might have been taken for the appendix vermiformis cavi: these processes had no cavities, having two flat sides only, and their colour was bluish inclining to black. The larger bulk of the hydatid was of a white colour, both externally and internally; upon the latter surface there were, here and there, stars of red lines, apparently blood-vessels.

The intestines were glued together with coagulable lymph, as is usual after fatal attacks of peritonitis. The kidneys were altered in shape, most probably from the pressure which they must have sustained. The liver was very much enlarged, but not tuberculated, and weighed nine pounds avoirdupois; the spleen was also very large, and weighed one pound. In the thorax there was no preternatural appearance of any importance. A little fluid was found in the right and left cavities, and some also within the pericardium.

We next examined the head, and discovered a small ulcerated opening in the integuments, covering the middle and upper part of the os frontis: this, we were informed, had existed nine or ten months, and that, whenever it closed, or the discharge from it was suppressed, the pain in the head was always very much increased. The integuments had acquired so firm an attachment to the subjacent parts, that a very unusual degree of trouble was experienced in separating them: when, however, effected, four or five tubercles, of the size and shape of a common horse-bean, were seen dispersed irregularly over the upper part of the head. These were cut through, and thick yellow pus was discharged from each, leaving the bone underneath carious. The longest portion of caries was in the os frontis, corresponding to the opening in the integuments first described: here it was of the size of a sixpence, and had penetrated nearly through the skull, which, upon measurement after it was sawn through, was in no part less than one-quarter of an inch in thickness. The upper part of the skull being removed, with its contiguous portion of dura mater, I found the adhesion so firm between this membrane and a large portion of bone, that I was unable to separate them by holding the skullcap in one hand, and pulling very forcibly the dura mater with the other; it required the assistance of a second person. This membrane appeared thickened, more opake than common, and was spotted in two or three places with coagulable lymph. I may also mention that, instead of the usual union to the brain by bloodvessels only, which enter the longitudinal sinus, there was a preternatural attachment to the superior surface of each hemisphere, two inches in length, in a direction from the anterior to the posterior part of the head: this attachment was so strong, that the scalpel was used freely to effect its separation. Mr. Charles Bell has the dura mater in preservation. In all other respects the brain seemed natural.

*Remarks.*—This curious case naturally produces some interrogatories, which I feel unable to answer. It has been asked if there was ever fluid in the abdominal cavity, independently of that contained in the hydatid cyst? There was certainly none after death; and, from the situation and connexion of the cyst, it being immediately contiguous to the inner surface of the abdominal muscles, and extending as low down as the pelvis, it must have been entered by the trocar, and the fluid discharged from it, the operation being performed in the linea alba, midway between the umbilicus and pubis; besides, the colour and consistence of the fluid being so like that found upon examining the dead body. Now, supposing that the openings were closed, (and of which there is pretty good evidence, from the accumulation of fluid within it and none without,) how is the diminution in the size of the abdomen previous to and at the term of death, to be accounted for? It may be answered, that the severe purging effected absorption; and this may be true; but I know not how far the hydatid structure is capable of allowing absorption, either during the life or death of this imperfect animal, so as materially to lessen its bulk. We know well that it possesses the power of increase and growth, and will sometimes multiply most abundantly.

I have been informed that ovarian dropsy has disappeared by absorption; but I believe this to be not an every-day occurrence.

The cause of the agonizing pains and disease of the cranial bones, though somewhat doubtful, was most probably a venereal one. All the evidence which I can collect from the husband and a female relative is, that Mrs. N., in the course of her practice, attended a woman in labour, and contracted a sore upon her thumb, where a small portion of cuticle had been torn off, and that this sore was supposed to be of a venereal nature. I cannot ascertain if mercury was exhibited, but am only informed that, by internal remedies and external applications, the sore healed in about six weeks, and that she frequently after had attacks of ulcerated sore-throat. A blistering-plaster was applied once, from which she obtained considerable relief: this might have been a common ulceration, or inflammation of the tonsils, from cold, &c. Two children were born dead, supposed from venereal contamination; but the last child, now five years old, and born one year before she complained of her head, is a fine healthy little fellow. The ulcer on the thumb occurred eleven years before her death, and seven years before she experienced pains in her head; also, the mercurial course, which I have since ascertained was instituted under the belief that syphilis was the cause of her sufferings, produced no alleviation.

P.S.—I should have stated that, during the severe purging which occurred a short time before death, the abdomen diminished very much in size.

*Mabledon-place; August 6th,* 1823.

